# Pathological Museum University of Texas

**Published:** 1893-07

**Authors:** Allen J. Smith

**Affiliations:** Professor of Pathology, University of Texas, Galveston, Texas


THE PATHOLOGICAL MUSEUM OF THE UNIVERSITY OF TEXAS. —The Medical Department of the University of Texas is exceedingly anxious to collect an many unique or typical examples of pathological processes as possible, for purposes of illustration in

*It is good sometimes to know something."

class and for preservation. The pathological department is prepared to receive any such specimens, and will gladly furnish any desired reports upon them. The specimens will be well preserved and labelled, and the name of the donor credited upon the label and in the catalogue of the museum. Physicians, and citizens of the State generally, are earnestly desired to forward to the Pathological Laboratory of the Medical Department of the University of Texas, Galveston, Texas, any specimens of medical interest, whether from the human body or not, such as tumors, malformations, diseased organs, calculi, specimens illustrating surgical injuries to bones or soft parts, specimens of parasites of man or beast, examples of poisonous reptiles, weapons—in short, anything which will serve to illustrate some point in medicine. Scientific curiosities bearing upon ethnology, the doctrine of evolution of species, comparative anatomy, all are most heartily welcome.

Any person who is willing to contribute to the development of the museum, may promptly obtain directions as to packing and transportation, by addressing

ALLEN J. SMITH, M. D.,
Professor of Pathology, University of Texas,
Galveston, Texas.

